# *Centella asiatica* modulates cancer cachexia associated inflammatory cytokines and cell death in leukaemic THP-1 cells and peripheral blood mononuclear cells (PBMC’s)

**DOI:** 10.1186/s12906-017-1865-2

**Published:** 2017-08-01

**Authors:** Dhaneshree Bestinee Naidoo, Anil Amichund Chuturgoon, Alisa Phulukdaree, Kanive Parashiva Guruprasad, Kapaettu Satyamoorthy, Vikash Sewram

**Affiliations:** 1 Discipline of Medical Biochemistry, Faculty of Health Sciences, Nelson Mandela School of Medicine, University of KwaZulu-Natal, Durban, 4013 South Africa; 2 Division of Biotechnology, School of Life Sciences, Manipal University, Planetarium Complex, Manipal, Karnataka 576 104 India; 3 African Cancer Institute, Faculty of Medicine and Health Sciences, Stellenbosch University, P.O. Box 241, Cape Town, 8000 South Africa; 4 Division of Health Systems and Public Health, Department of Global Health, Faculty of Medicine and Health Sciences, Stellenbosch University, P.O. Box 241, Cape Town, 8000 South Africa

**Keywords:** Cancer, Cachexia, Cytokines, Apoptosis, *Centella asiatica*

## Abstract

**Background:**

Cancer cachexia is associated with increased pro-inflammatory cytokine levels. *Centella asiatica* (*C. asiatica*) possesses antioxidant, anti-inflammatory and anti-tumour potential. We investigated the modulation of antioxidants, cytokines and cell death by *C. asiatica* ethanolic leaf extract (C_LE_) in leukaemic THP-1 cells and normal peripheral blood mononuclear cells (PBMC’s).

**Methods:**

Cytotoxcity of C_LE_ was determined at 24 and 72 h (h). Oxidant scavenging activity of C_LE_ was evaluated using the 2, 2-diphenyl-1 picrylhydrazyl (DPPH) assay. Glutathione (GSH) levels, caspase (−8, −9, −3/7) activities and adenosine triphosphate (ATP) levels (Luminometry) were then assayed. The levels of tumour necrosis factor-α (TNF-α), interleukin (IL)-6, IL-1β and IL-10 were also assessed using enzyme-linked immunosorbant assay.

**Results:**

C_LE_ decreased PBMC viability between 33.25–74.55% (24 h: 0.2–0.8 mg/ml C_LE_ and 72 h: 0.4–0.8 mg/ml C_LE_) and THP-1 viability by 28.404% (72 h: 0.8 mg/ml C_LE_) (*p* < 0.0001). Oxidant scavenging activity was increased by C_LE_ (0.05–0.8 mg/ml) (*p* < 0.0001). PBMC TNF-α and IL-10 levels were decreased by C_LE_ (0.05–0.8 mg/ml) (*p* < 0.0001). However, PBMC IL-6 and IL-1β concentrations were increased at 0.05–0.2 mg/ml C_LE_ but decreased at 0.4 mg/ml C_LE_ (*p* < 0.0001). In THP-1 cells, C_LE_ (0.2–0.8 mg/ml) decreased IL-1β and IL-6 whereas increased IL-10 levels (*p* < 0.0001). In both cell lines, C_LE_ (0.05–0.2 mg/ml, 24 and 72 h) increased GSH concentrations (*p* < 0.0001). At 24 h, caspase (−9, −3/7) activities was increased by C_LE_ (0.05–0.8 mg/ml) in PBMC’s whereas decreased by C_LE_ (0.2–0.4 mg/ml) in THP-1 cells (*p* < 0.0001). At 72 h, C_LE_ (0.05–0.8 mg/ml) decreased caspase (−9, −3/7) activities and ATP levels in both cell lines (*p* < 0.0001).

**Conclusion:**

In PBMC’s and THP-1 cells, C_LE_ proved to effectively modulate antioxidant activity, inflammatory cytokines and cell death. In THP-1 cells, C_LE_ decreased pro-inflammatory cytokine levels whereas it increased anti-inflammatory cytokine levels which may alleviate cancer cachexia.

## Background

The role of inflammation in carcinogenesis has been extensively documented [1]. Although inflammatory responses have shown beneficial effects in tissue repair and pathogen elimination [1, 2], chronic inflammation has been implicated in tumour initiation, promotion and progression [3]. During ideal conditions, the host-mediated anti-tumour activity combats the tumour-mediated immunosuppressive activity and cancerous cells are sentenced to cell death [3]. In the event that the host anti-tumour activity is weakened/inadequate, the persistent and enhanced pro-inflammatory tumour microenvironment will facilitate tumour development, invasion, angiogenesis and metastasis [3].

Many malignancies are associated with the cachectic syndrome [4], a disorder characterised by abnormal weight loss [5] due to adipose tissue (85%) and skeletal muscle (75%) depletion [6]. The enzyme lipoprotein lipase (LPL) hydrolyses fatty acids (FA’s) and transports FA’s into adipose tissue for triacylglycerol (TAG) production, whereas hormone sensitive lipase (HSL) breaks down TAG’s into FA’s and glycerol [6]. Studies have revealed that decreased serum LPL levels/activity [7, 8] and increased HSL levels/activity are associated with cachexia [9]. Additionally, increased proteolysis and decreased proteogenesis have been reported in cachectic patients [10]. The ATP-ubiquitin-dependent proteolytic pathway has been shown to be responsible for the excessive proteolysis seen in cancer cachexia [11].

Oxidative stress, inflammatory cytokines and apoptosis play a pivotal role in the initiation and development of cancer cachexia [12]. Inflammatory cytokine production is increased by lipopolysaccharide (LPS) potently stimulating macrophages [13]. The LPS signal is transduced by LPS binding to LPS binding protein, delivered to CD14 and transferred to Toll like receptor-4 [14]. This subsequently activates nuclear factor kappa B (NF-κB), which regulates the transcription of genes associated with inflammation, proliferation, invasion, angiogenesis and apoptosis [1, 15–17]. Previously, IL-1 [18], IL-6 (mice) [19] and TNF-α (rat, mouse and guinea pigs) [20] were shown to decrease LPL activity in adipose tissue. Decreased LPL activity reduces the uptake of exogenous lipids by adipose tissue [20], which decreases lipogenesis. Additionally, previous literature showed that TNF-α increased ubiquitin (concentrations and mRNA), while IL-6 increased the 26S proteasome and cathepsin activities, suggesting the activation of proteolytic pathways [21–24]. The activation of proteolytic pathways causes extensive muscle wasting through proteolysis. Taken together, an excessive increase in pro-inflammatory cytokine levels may increase tumour immunosuppressive activity [3], as well as tissue wasting [6].

Oxidative stress has been associated with tumour initiation, inflammation [2, 3] and muscle wasting [25]. However, antioxidants have been shown to decrease muscle wasting by neutralizing reactive oxygen species (ROS) [1, 25]. Elevated ROS levels activate apoptotic pathways, ultimately activating caspase-3 [26]. The activation of caspase-3 plays an important role in the execution of apoptosis as well as muscle proteolysis [27]. Additionally, in weight-losing upper gastrointestinal tract cancer patients, deoxyribonucleic acid (DNA) fragmentation and poly (ADP-ribose) polymerase (PARP) cleavage were increased, whereas MyoD protein was decreased [6], suggesting increased apoptosis and decreased muscle replenishment.

There is a constant need for alternative traditional medicines to improve the prognosis of cancer patients and prevent chemotherapy and radiotherapy induced discomfort. The tropical medicinal plant *Centella asiatica* (Linnaeus) Urban (*C. asiatica*) is native to India, China, and South Africa [28]. It belongs to the Apiaceae family and is commonly referred to as Gotu kola, Asiatic pennywort and Tiger herb [28]. *C. asiatica* is widely used in Ayurvedic and Chinese traditional medicines due to its various medicinal properties. These properties include its hepato-protective, cardio-protective, anti-diabetic, antioxidant, anti-inflammatory and anti-tumour potential [28]. The major active compounds in *C. asiatica* are triterpene saponosides such as asiatic acid, madecassic acid and asiaticoside [28]. *C. asiatica* also contains flavonoid derivatives, vitamins, minerals, polysaccharides, sterols and phenolic acids [28]. *C. asiatica* has previously been used in treatment of inflammation due to its promising anti-inflammatory effects [29, 30]. Additionally, *C. asiatica* extracts have demonstrated high antioxidant [31, 32] and anti-proliferative activity in many cancerous cell lines [33].

There is a need for the discovery of an inexpensive cancer cachectic treatment. The ability of a plant extract to regulate inflammatory cytokines and cell death may elevate cancerous cell death and diminish tissue wasting. We investigated the potential of a *C. asiatica* ethanolic leaf extract (C_LE_) to modulate inflammatory cytokines, antioxidants and cell death in leukaemic THP-1 cells and normal peripheral blood mononuclear cells (PBMC’s).

## Methods

### Materials

*C. asiatica* leaves were collected on the 7th of March 2011 (collectors number: Immelman 411) from the Eastern Cape [Langeni forest, roadside (S31°28.135′, E28°32.681′)], South Africa (SA) and identified by Dr. Kathleen Immelman from the Department of Botany at the Walter Sisulu University, SA. Voucher specimens were deposited at the KEI herbarium (13979). The THP-1 cells were obtained from American Type Culture Collection (ATCC, University Boulevard Manassas, Virginia, USA). RPMI-1640 and BD OptEIA enzyme-linked immunosorbant assay (ELISA) cytokine kits were purchased from The Scientific Group (Johannesburg, SA). Foetal calf serum (FCS) and Pen/Strep Amphotericin B (PSF) were acquired from Whitehead Scientific (Cape Town, SA). Dimethyl sulphoxide (DMSO) was purchased from Merck (Johannesburg, SA). Histopaque-1077, LPS and 2, 2-diphenyl-1 picrylhydrazyl (DPPH) were purchased from Sigma (Aston Manor, SA). The 4-[3-(4-iodophenyl)-2-(4-nitrophenyl)-2H-5-tetrazolio]-1,3-benzene disulphonate (WST-1) cell proliferation reagent was purchased from Roche (Johannesburg, SA). Promega (Madison, USA) supplied the caspase (−3/7, −8, −9), adenosine triphosphate (ATP) and glutathione (GSH) kits.

### Plant description and extraction

The plants official name is *Centella asiatica* (L.) Urb and has been confirmed by using the plant list [34]. The English name is Tiger herb. *C. asiatica* leaves were dried and milled. Ethanol (200–350 ml) was added to milled plant material (10–30 g) and extracted overnight by shaking (4×g, 37 °C). Ethanol extracts were filtered, rotor evaporated, dried (37 °C) and stored (4 °C).

### The 2, 2-diphenyl-1 picrylhydrazyl assay

C_LE_ (0.05–0.8 mg/ml) and butylated hydroxytoluene (BHT) (60–300 μM) dilutions were prepared in methanol (99.5% and grade AR). A 50 μM DPPH solution was prepared from a stock solution of 0.135 mM DPPH in methanol. C_LE_, BHT dilutions and methanol (1 ml, triplicate tubes) were aliquoted into 15 ml polypropylene tubes, followed by the 50 μM DPPH solution (1 ml). Reaction mixtures were vortexed and incubated (room temperature (RT) for 30 min (min)) in the dark. Absorbance of samples was read at 517 nm using a Varine Cary 50 UV-visible spectrophotometer (McKinley Scientific, New Jersey, US).

### Isolation of peripheral blood mononuclear cells

Buffy coats containing PBMC’s were obtained from the South African National Blood Service (2011/09). PBMC’s were extracted by differential centrifugation. Buffy coats (5 ml) were layered onto equivolume histopaque-1077 (5 ml) in 15 ml polypropylene tubes and centrifuged (400×g, 21 °C for 30 min). After centrifugation, the PBMC’s were transferred to sterile 15 ml polypropylene tubes, phosphate buffered saline (PBS) was added (0.1 M, 10 ml) and tubes were centrifuged (400×g, 21 °C, 15 min). Cell density of isolated PBMC’s was adjusted (1 × 10^6^ cells/ml) using the trypan blue exclusion test and cryo-preserved (10% FCS, 10% DMSO) using a NELGENE cryo freezing container and stored at −80 °C.

### Tissue culture

THP-1 cells were grown in the appropriate tissue culture conditions in a 75 cm^3^ tissue culture flask (37 °C, 5% CO_2_). The growth media comprised of RPMI-1640, FCS (10%) and PS (2%). Cells were thawed, seeded into a 75 cm^3^ tissue culture flask at a concentration of 3 × 10^5^ cells/ml and incubated (37 °C, 5% CO_2_). THP-1 cells were allowed to grow for 2–3 days before the cells were centrifuged (162×g, 10 min) and re-suspended in fresh growth media. The number of cells should not exceed 8 × 10^5^ cells/ml, therefore the cells/ml was quantified daily by trypan blue staining. Once the cell count reached 8 × 10^5^ cells/ml the THP-1 cells were split/ diluted to 3 × 10^5^ cells/ml with media and incubated. Subsequent experiments were conducted once the cell numbers were sufficient.

### Cell viability assay

Cytotoxicity of C_LE_ in PBMC’s and THP-1 cells was measured using the WST-1 assay (Roche, Johannesburg, SA). PBMC and THP-1 cells (10,000 cells/well, 96-well plate, in triplicate wells) were stimulated with LPS (20 μg/ml, 37 °C, 5% CO_2_, 4 h (h)) before exposure to C_LE_ (0.05–0.8 mg/ml) for 24 and 72 h (37 °C, 5% CO_2_). Similarly, controls received media containing DMSO (0.2%). Thereafter, plates were centrifuged (162×g, 10 min), supernatant removed, cell pellets re-suspended in growth media (100 μl/well), WST-1 reagent (10 μl/well) added and plates incubated (37 °C, 5%, CO_2_, 3 h). Optical density was measured at 450 nm (620 nm reference wavelength) with a BIO-TEK μQuant spectrophotometer (Analytical and Diagnostic Products, SA). This experiment was conducted independently on three occasions.

### Stimulation and treatment of cells

PBMC’s and THP-1 cells (1 × 10^5^ cells/ml) were transferred into 24-well plates, stimulated with LPS (20 μg/ml, 37 °C, 5% CO_2_, 4 h) before exposure to C_LE_ (0.05–0.8 mg/ml) for 24 h (TNF-α) and 72 h (IL-1β, IL-6, IL-10) (37 °C, 5% CO_2_). After incubation, plates were centrifuged (162×g, 10 min) and supernatant was collected and stored (−80 °C) for cytokine analysis. Cell pellets were used to conduct the caspase (−8, −9, −3/7) activity, ATP and GSH assays. The experiments were conducted independently (twice for all subsequent assays).

### Quantification of cytokines

Cytokine levels were estimated using the BD OptEIA ELISA kits (The Scientific Group, SA) and the procedure was followed as per the instruction manual. ELISA plates were coated with capture antibody overnight (100 μl/well, 4 °C). Thereafter, plates were washed (3×) with wash buffer and blocked with assay diluent (200 μl/well, 1 h, RT). Standard solutions were prepared by diluting a stock solution [TNF-α, IL-10 (500 pg/ml), IL-6 (300 pg/ml), IL-1β (250 pg/ml)] serially [TNF-α, IL-10 (500–7.8 pg/ml), IL-6 (300–4.7 pg/ml), IL-1β (250–3.9 pg/ml)]. Plates were washed (3×), standards and samples (100 μl/well, triplicate wells) were aliquoted into appropriate wells and plates were incubated (2 h, RT). Plates were washed (5×), working detector (100 μl/well) added and plates incubated (1 h, RT). The plates were washed (7×), substrate solution (100 μl/well) added and plates were incubated (30 min, RT) in the dark. Finally, stop solution (50 μl/well) was added and the absorbance was read at 450 nm (570 nm reference wavelength) with a Multiskan FC micro-plate reader (Thermo Scientific). Cytokine concentrations were calculated by extrapolation from a standard curve.

### Glutathione assay

The GSH-Glo™ assay (Promega, Madison, WI, USA) was used to measure GSH levels. Standard GSH solutions were prepared by diluting a 5 mM stock solution serially (1.56–50 μM) and PBS (0.1 M) was the standard blank. Cells (50 μl/well, 2 × 10^5^ cells/ml) and standards were added into an opaque 96-well plate (duplicate wells), followed by GSH-Glo™ reagent (25 μl/well) and allowed to incubate (30 min, RT) in the dark. Subsequently, luciferin detection reagent (50 μl/well) was added and plates incubated (15 min, RT) in the dark. The absorbance was read on a Modulus™ microplate luminometer (Turner Biosystems, Sunnyvale, USA) and GSH concentrations were calculated by extrapolation from a standard curve.

### Caspase and ATP assays

Caspase activity and ATP levels were determined using the Caspase-Glo®-3/7, −8, −9 and ATP assay kits (Promega, Madison, WI, USA). Caspase-Glo®-3/7, −8, −9 and ATP reagents were reconstituted according to the manufacturer’s instructions. Cells (100 μl, 2 × 10^5^ cells/ml) were added into duplicate wells of a microtitre plate for each assay, thereafter caspase −3/7, −8, −9 and ATP reagents (100 μl/well) were added into appropriate wells. The plate was incubated (30 min, RT) in the dark. Luminescence was measured on a Modulus™ microplate luminometer (Turner BioSystems) and expressed as relative light units (RLU).

### Statistical analysis

Statistical analysis was performed using the STATA and GraphPad Prism (v5) statistical analysis software. The one-way analysis of variance (ANOVA) was used to make comparisons between groups, followed by the Tukey multiple comparisons test, with *p* < 0.05 indicating significant results.

## Results

### The oxidant scavenging potential of C_LE_

**Fig. 1:**
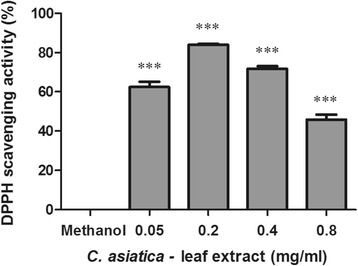
Percentage DPPH scavenging activity of C_LE_ (Values expressed as mean ± SD, ^***^ *p* < 0.0001 compared to control)

The oxidant scavenging activity of C_LE_ using the DPPH assay is shown in Fig. 1. C_LE_ (0.05–0.8 mg/ml) significantly increased DPPH scavenging activity by approximately 45–84% (Fig. 1, *p* < 0.0001).

### The in vitro cytotoxicity of C_LE_

**Fig. 2:**
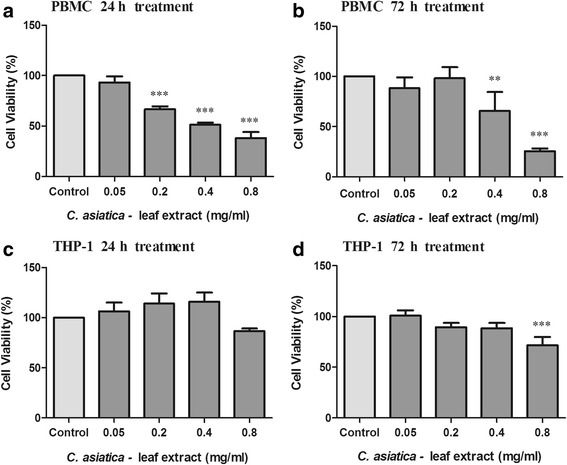
Cell viability of PBMC (**a** – 24 h, **b** – 72 h) and THP-1 (**c** – 24 h, **d** – 72 h) cells treated with C_LE_ for 24 and 72 h (Values expressed as mean ± SD, ^**^ *p* < 0.001, ^***^ *p* < 0.0001 compared to the control)

The WST-1 assay was used to determine cell viability of THP-1 cells and PBMC’s after treatment with C_LE_ (Fig. 2). At 24 h, C_LE_ (0.2–0.8 mg/ml) dose dependently decreased PBMC viability by 33.25–61.85% (Fig. 2a, *p* < 0.0001), whereas THP-1 viability was not significantly altered as compared to the control (Fig. 2c, *p* = 0.0003). At 72 h, C_LE_ decreased both PBMC (Fig. 2b, 34.268–74.547%) and THP-1 (Fig. 2d, czmg/ml respectively as compared to the control (*p* < 0.0001), suggesting that PBMC’s are more sensitive to C_LE_ treatment than THP-1 cells.

### The immune suppressive properties of C_LE_

**Fig. 3:**
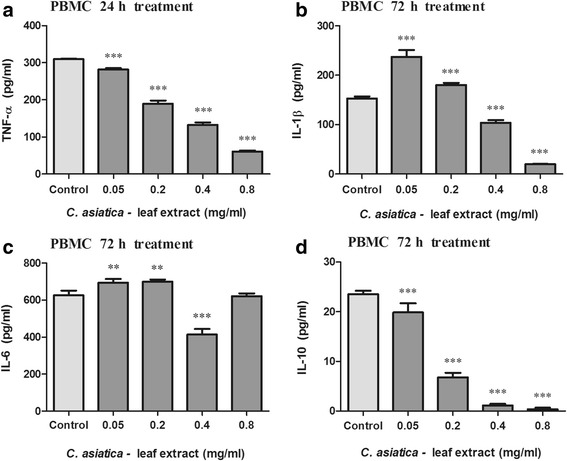
Concentration of TNF-α (**a**), IL-1β (**b**), IL-6 (**c**) and IL-10 (**d**) in C_LE_ treated PBMC’s (Values expressed as mean ± SD, ^*^ *p* < 0.01, ^***^ *p* < 0.0001, compared to the control)

**Fig. 4:**
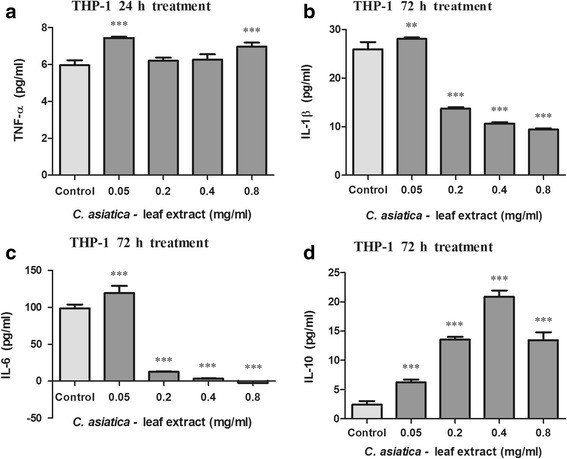
Concentration of TNF-α (**a**), IL-1β (**b**), IL-6 (**c**) and IL-10 (**d**) in C_LE_ treated THP-1 cells (Values expressed as mean ± SD, ^**^ *p* < 0.001, ^***^ *p* < 0.0001 compared to the control)

C_LE_ altered cytokine levels in PBMC’s and THP-1 cells which are shown in Figs. 3 and 4 respectively. The levels of TNF-α, IL-1β, IL-6 and IL-10 produced in LPS stimulated PBMC’s was 309.60, 152.83, 626.33 and 23.55 pg/ml respectively. C_LE_ (0.05–0.2 mg/ml) increased PBMC IL-1β and IL-6 concentrations relative to the control (Fig. 3b–c, *p* < 0.0001). In PBMC’s, TNF-α, IL-1β and IL-6 concentrations were decreased at 0.05–0.8 mg/ml C_LE_, 0.4–0.8 mg/ml C_LE_ and 0.4 mg/ml C_LE_ respectively as compared to the control (Fig. 3a–c, *p* < 0.0001). The levels of TNF-α, IL-1β, IL-6 and IL-10 produced in LPS stimulated THP-1 cells was 5.96, 25.92, 98.63, and 2.46 pg/ml respectively. TNF-α concentration in THP-1 cells was increased by C_LE_ (0.05, 0.8 mg/ml, Fig. 4a, *p* < 0.0001) relative to the control. In THP-1 cells, IL-1β and IL-6 concentrations were increased by 0.05 mg/ml C_LE_ whereas decreased by 0.2–0.8 mg/ml C_LE_ as compared to the control (Fig. 4b–c, *p* < 0.0001). Concentration of the anti-inflammatory cytokine, IL-10 was decreased in PBMC’s while increased in THP-1 cells by C_LE_ (0.05–0.8 mg/ml) relative to the control (Figs. 3d and 4d, *p* < 0.0001).

### The antioxidant potential of C_LE_

The endogenous antioxidant activity of C_LE_ was determined by measuring GSH levels in both cell lines (Table 1). At 24 h, GSH levels in PBMC’s were increased by 0.05–0.2 mg/ml C_LE_ but decreased by 0.4–0.8 mg/ml C_LE_ relative to the control (Table 1, *p* < 0.0001). In THP-1 cells, C_LE_ (0.05–0.8 mg/ml) increased GSH levels as compared to the control (Table 1, 24 h, *p* < 0.0001). At 24 h, GSH concentrations were increased to a greater extent in THP-1 cells (0.068–3.890 μM) than PBMC’s (0.191–1.746 μM). At 72 h, C_LE_ (0.05–0.8 mg/ml) increased GSH concentrations in PBMC’s and THP-1 cells by 1.13–5.91 μM and 0.12–0.19 μM respectively as compared to the control (Table 1, *p* < 0.0001). Notably, C_LE_ increased GSH levels to a greater extent in PBMC’s as compared to THP-1 cells at 72 h.

**Table 1:** Glutathione levels in C_LE_ treated PBMC’s and THP-1 cells

	Glutathione (μM)			
C_LE_ (mg/ml)	24 h treatment		72 h treatment	
	PBMC	THP-1	PBMC	THP-1
Control	1.238 ± 0.007	1.713 ± 0.002	3.842 ± 0.009	1.449 ± 0.002
0.05	1.429 ± 0.007^***^	4.125 ± 0.004^***^	9.138 ± 0.082^***^	1.576 ± 0.007^***^
0.2	2.984 ± 0.004^***^	5.603 ± 0.004^***^	4.972 ± 0.003^***^	1.568 ± 0.007^***^
0.4	0.959 ± 0.002^***^	1.781 ± 0.002^***^	5.534 ± 0.011^***^	1.610 ± 0.009^***^
0.8	1.073 ± 0.015^***^	2.495 ± 0.005^***^	9.749 ± 0.015^***^	1.634 ± 0.004^***^

(Values expressed as mean ± SD, ^***^ *p* < 0.0001, compared to the control)

### C_LE_ modulates caspase (−8, −9, −3/7) activities and ATP levels

Luminometry assays were used to determine caspase activity and ATP levels in THP-1 cells and PBMC’s after treatment with C_LE_. The pro-apoptotic effect of C_LE_ in PBMC’s treated for 24 h is shown in Table 2. At 24 h, PBMC caspase-8 activity was increased by 0.05–0.2 mg/ml C_LE,_ whereas decreased by 0.4–0.8 mg/ml C_LE_ as compared to the control (Table 2, *p* < 0.0001). C_LE_ (0.05–0.8 mg/ml, 24 h) increased PBMC caspase −9 and −3/7 activities relative to the control (Table 2, *p* < 0.0001). Increased caspase activity led to the initiation and execution of PBMC apoptosis at 24 h. The PBMC ATP levels were increased by 0.4 mg/ml C_LE,_ whereas decreased by 0.05, 0.2 and 0.8 mg/ml C_LE_ (Table 2, *p* < 0.0001).

**Table 2:** Modulation of caspase (−8, −9, −3/7) activities and ATP levels in 24 h C_LE_ treated PBMC’s

C_LE_ (mg/ml)	Caspase-8 (RLU × 10^5^)	Caspase-9 (RLU × 10^5^)	Caspase-3/7 (RLU × 10^5^)	ATP (RLU × 10^5^)
Control	0.146 ± 0.001	0.265 ± 0.002	5.861 ± 0.028	3.486 ± 0.011
0.05	0.176 ± 0.001^***^	0.293 ± 0.001^***^	6.066 ± 0.032	3.168 ± 0.006^***^
0.2	0.256 ± 0.003^***^	0.364 ± 0.002^***^	6.264 ± 0.031^**^	3.074 ± 0.002^***^
0.4	0.135 ± 0.001^***^	0.397 ± 0.0003^***^	16.407 ± 0.263^***^	4.180 ± 0.013^***^
0.8	0.101 ± 0.001^***^	0.307 ± 0.0004^***^	6.331 ± 0.007^***^	0.796 ± 0.002^***^

(Values expressed as mean ± SD, ^**^ *p* < 0.001, ^***^ *p* < 0.0001 compared to the control)

C_LE_ pro-apoptotic effects in THP-1 cells treated for 24 h is shown in Table 3. At 24 h, C_LE_ (0.05–0.8 mg/ml) increased THP-1 caspase-8 activity as compared to the control (Table 3, *p* < 0.0001). In THP-1 cells, caspase-9 activity and ATP levels were decreased by 0.05–0.4 mg/ml C_LE,_ whereas increased by 0.8 mg/ml C_LE_ relative to the control (Table 3, 24 h, *p* < 0.0001). The THP-1 caspase-3/7 activity was decreased by 0.2–0.4 mg/ml C_LE,_ whereas increased by 0.05 and 0.8 mg/ml C_LE_ as compared to the control (Table 3, 24 h, *p* < 0.0001). THP-1 caspase (−8, −9, −3/7) activities was increased by 0.8 mg/ml C_LE_, suggesting an increased initiation and execution of THP-1 apoptosis.

**Table 3:** Modulation of caspase (−8, −9, −3/7) activities and ATP levels in 24 h C_LE_ treated THP-1 cells

C_LE_ (mg/ml)	Caspase-8 (RLU × 10^5^)	Caspase-9 (RLU × 10^5^)	Caspase-3/7 (RLU × 10^5^)	ATP (RLU × 10^5^)
Control	8.517 ± 0.001	1.933 ± 0.012	9.980 ± 0.008	17.551 ± 0.088
0.05	11.494 ± 0.006^***^	0.415 ± 0.002^***^	10.348 ± 0.218^**^	12.507 ± 0.398^***^
0.2	18.909 ± 0.085^***^	0.675 ± 0.001^***^	3.974 ± 0.001^***^	15.586 ± 0.215^***^
0.4	12.276 ± 0.028^***^	1.119 ± 0.003^***^	4.046 ± 0.033^***^	3.948 ± 0.042^***^
0.8	16.191 ± 0.013^***^	2.261 ± 0.002^***^	18.189 ± 0.104^***^	19.496 ± 0.267^***^

(Values expressed as mean ± SD, ^**^ *p* < 0.001, ^***^ *p* < 0.0001 compared to the control)

The pro-apoptotic effect of C_LE_ in PBMC’s treated for 72 h is shown in Table 4. At 72 h, PBMC caspase-8 activity was increased by 0.4 mg/ml C_LE,_ whereas decreased by 0.05, 0.2, 0.8 mg/ml C_LE_ relative to the control (Table 4, *p* < 0.0001). C_LE_ (0.05–0.8 mg/ml) decreased PBMC caspase (−9, −3/7) activities and ATP levels as compared to the control (Table 4, 72 h, *p* < 0.0001). Decreased PBMC caspase activity suggests a decrease in PBMC apoptotic cell death.

**Table 4:** Modulation of caspase (−8, −9, −3/7) activities and ATP levels in 72 h C_LE_ treated PBMC’s

C_LE_ (mg/ml)	Caspase-8 (RLU × 10^5^)	Caspase-9 (RLU × 10^5^)	Caspase-3/7 (RLU × 10^5^)	ATP (RLU × 10^5^)
Control	30.688 ± 0.006	83.054 ± 0.009	132.624 ± 0.118	14.567 ± 0.184
0.05	21.726 ± 0.015^***^	56.070 ± 0.003^***^	128.471 ± 0.253^***^	4.061 ± 0.014^***^
0.2	10.436 ± 0.021^***^	25.014 ± 0.007^***^	57.946 ± 0.024^***^	2.343 ± 0.029^***^
0.4	42.625 ± 0.003^***^	11.887 ± 0.005^***^	35.842 ± 0.036^***^	0.855 ± 0.002^***^
0.8	14.157 ± 0.045^***^	32.499 ± 0.288^***^	43.376 ± 0.028^***^	3.117 ± 0.007^***^

(Values expressed as mean ± SD, ^***^ *p* < 0.0001 compared to the control)

C_LE_ pro-apoptotic effects in THP-1 cells treated for 72 h is shown in Table 5. At 72 h, THP-1 caspase-8 activity was increased by 0.4 mg/ml C_LE_ whereas decreased by 0.05, 0.2, 0.8 mg/ml C_LE_ relative to the control (Table 5, *p* < 0.0001). C_LE_ (0.05–0.8 mg/ml) decreased THP-1 caspase (−9, −3/7) activities and ATP levels as compared to the control (Table 5, 72 h, *p* < 0.0001). Decreased THP-1 caspase activity suggests a decrease in THP-1 apoptotic cell death.

**Table 5:** Modulation of caspase (−8, −9, −3/7) activities and ATP levels in 72 h C_LE_ treated THP-1 cells

C_LE_ (mg/ml)	Caspase-8 (RLU × 10^5^)	Caspase-9 (RLU × 10^5^)	Caspase-3/7 (RLU × 10^5^)	ATP (RLU × 10^5^)
Control	1.068 ± 0.002	6.694 ± 0.002	8.218 ± 0.002	4.552 ± 0.029
0.05	1.021 ± 0.001^**^	6.343 ± 0.009^***^	6.293 ± 0.001^***^	4.252 ± 0.039^***^
0.2	0.972 ± 0.0003^***^	5.442 ± 0.034^***^	4.954 ± 0.002^***^	3.852 ± 0.039^***^
0.4	11.246 ± 0.034^***^	4.271 ± 0.001^***^	3.596 ± 0.005^***^	3.013 ± 0.005^***^
0.8	0.286 ± 0.0001^***^	1.720 ± 0.001^***^	0.497 ± 0.001^***^	1.065 ± 0.011^***^

(Values expressed as mean ± SD, ^**^ *p* < 0.001, ^***^ *p* < 0.0001 compared to the control)

## Discussion

Cancer and cachexia have been associated with increased levels of oxidative stress, pro-inflammatory cytokines and apoptosis [6, 27]. The medicinal plant, *C. asiatica* possesses anti-inflammatory [29] and anti-tumor activity [35], which can be beneficial in the treatment of cancer cachexia.

Previously, Zainol et al. (2003) reported that *C. asiatica* possessed antioxidant potential, possibly associated with phenolic compounds [36]. The DPPH assay revealed that C_LE_ has oxidant scavenging potential ranging between 45 and 84% at 0.05–0.8 mg/ml C_LE_. ROS plays a pivotal role in tumour initiation, inflammation, protein degradation and apoptosis. The significant oxidant scavenging potential of C_LE_ may decrease inflammatory cytokine levels and ROS induced apoptosis.

At 24 h, C_LE_ dose dependently decreased PBMC viability, whereas THP-1 viability remained unchanged. However, at 72 h, C_LE_ significantly decreased both PBMC and THP-1 viability. *C. asiatica* derived compounds, asiatic acid and asiticoside, were shown to reduce RAW 264.7 cell viability (120 μM, 24 h) by 82% and 71% respectively [37]. Additionally, *C. asiatica* extracts inhibited breast (MCF-7) and liver (HepG2) cancer cell proliferation [33, 38], indicating our data on C_LE_ cytotoxicity is in agreement with previous studies.

Inflammatory cytokines play an essential role in tumourgenesis and the cachectic syndrome [6]. Previously, Punturee et al. (2004) reported that *C. asiatica* ethanolic extract modulated/suppressed TNF-α production in mouse macrophages [39]. Our results also show that C_LE_ decreased TNF-α concentration in PBMC’s. Yun et al. (2008) reported that the pre-treatment of RAW264.7 cells with asiatic acid significantly reduced IL-6 production with minimal effects on TNF-α and IL-1β levels [37]. Our findings, however, suggest that C_LE_ modulates pro-inflammatory cytokine levels. In both PBMC’s and THP-1 cells, IL-1β and IL-6 levels were increased by the lower 0.05 mg/ml C_LE_ concentration but decreased at the higher 0.4 mg/ml C_LE_ concentration. Pro-inflammatory cytokines, over a chronic time period, stimulate the production of genotoxic molecules [nitric oxide (NO), ROS] and tumour progression by promoting angiogenesis and metastasis [1, 3]. Previous literature has shown that IL-1 stimulates malignant cell growth and invasiveness [3]. In addition, IL-6 exerts its tumour proliferative and anti-apoptotic potential by targeting genes involved in cell cycle progression and the suppression of apoptosis [3]. The ability of C_LE_ to increase pro-inflammatory cytokines such as IL-1β in PBMC’s may aid in cancerous cell elimination through increased host anti-tumour activity. Conversely, in THP-1 cells, the decrease in IL-6 and IL-1β concentrations by C_LE_ may diminish cytokine induced tumour immunosuppressive activity and cancer progression.

With regard to the cachectic syndrome, TNF-α inhibits the production of LPL and reduces the rate of LPL gene transcription [40–42], thereby preventing the formation of new lipid stores while stimulating HSL and increasing lipolysis [43]. In adipose tissue (in vivo), IL-6 decreased LPL activity leading to tissue wasting in cachectic individuals [19]. The potential of C_LE_ (0.4 mg/ml) to decrease IL-6 and IL-1β concentrations in PBMC’s and THP-1 cells suggests a decrease in LPL inhibition and HSL stimulation, thus contributing to lipogenesis maintenance and minimal lipolysis. IL-6 and TNF-α further contribute to cachexia by stimulating muscle catabolism through the activation of the ubiquitin-proteasome pathway [21, 22, 44]. Furthermore, pro-inflammatory cytokines activate NF-κB which regulates the expression of genes involved in the suppression of tumour apoptosis, stimulation of tumour cell cycle progression and enhancement of inflammatory mediators [1, 3]. Taken together, NF-κB promotes tumour progression, invasion, angiogenesis and metastasis [1, 3]. In cachexia, NF-κB activation induces ubiquitin–proteasome pathway activity and suppresses MyoD expression [45], thereby increasing proteolysis and decreasing muscle replenishment [46]. By decreasing IL-6 and IL-1β concentrations in PBMC’s and THP-1 cells, C_LE_ (0.4 mg/ml) may prevent excessive activation of NF-κB and proteasome pathways, ultimately decreasing proteolysis. Taken together, C_LE_ may be able to decrease tissue wasting through the down regulation of pro-inflammatory cytokine levels.

The immunosuppressive and anti-inflammatory cytokine IL-10, inhibits tumour development, tumour progression, modulates apoptosis and suppresses angiogenesis during tumour regression [1, 3]. Additionally, IL-10 inhibits NF-κB activation and subsequently inhibits pro-inflammatory cytokine production (TNF-α, and IL-6) [3]. With regard to tissue wasting, increased IL-10 levels in colon 26- bearing mice was reported to reverse the cachectic syndrome [47]. The decreased PBMC IL-10 concentration may be due to IL-10 combating increased pro-inflammatory cytokine levels (IL-6 and IL-1β). In THP-1 cells, the potential of C_LE_ to increase IL-10 levels will facilitate a decrease in pro-inflammatory cytokine levels, a decrease in malignant cell progression and possibly alleviate the cancer cachectic syndrome.

GSH, a potent antioxidant [48], effectively scavenges ROS both directly and indirectly [49]. In PBMC’s and THP-1 cells, C_LE_ increased GSH concentrations. At 72 h, C_LE_ (0.4 mg/ml) increased GSH levels more significantly in PBMC’s (1.45-fold) than THP-1 cells (1.11-fold). This suggests that C_LE_ induces a higher antioxidant defense in normal PBMC’s than cancerous THP-1 cells at 72 h.

Apoptosis is a tightly regulated process involving a number of check points before an irreversible point is reached [50]. The extrinsic (death receptors) and intrinsic (mitochondria) pathways are the two main apoptotic pathways [26]. Activation of initiator caspases (−8, −9) leads to the activation of execution caspase-3/7 resulting in activation of cytoplasmic endonucleases [26].

Previous studies reported that asiatic acid decreased cell viability, induced apoptosis and DNA fragmentation [51, 52]. In PBMC’s, C_LE_ (0.4–0.8 mg/ml, 24 h) decreased caspase-8 activity. An increase in TNF-α levels initiates the extrinsic apoptotic pathway subsequently activating caspase-8. However, C_LE_ decreased PBMC TNF-α levels which may have contributed to the decreased caspase-8 activity. At 24 h, C_LE_ increased PBMC caspase (−8 (0.05–0.2 mg/ml), −9, −3/7 (0.05–0.8 mg/ml)) activities, suggesting the activation of the extrinsic and intrinsic apoptotic pathways. GSH regulates apoptosis by preventing ROS accumulation [53]. Previous studies have demonstrated that elevated GSH levels have been associated with resistance to apoptosis [54, 55]. In PBMC’s, the decrease in GSH levels and the increase in caspase (−9, −3/7) activities by C_LE_ (0.4–0.8 mg/ml, 24 h) may have increased apoptosis ultimately decreasing PBMC cell viability. In THP-1 cells, C_LE_ (0.05–0.4 mg/ml) increased caspase-8 activity and decreased caspase-9 activity, suggesting initiation of apoptosis through the extrinsic pathway (24 h). In C_LE_ treated THP-1 cells, the decreased caspase-9 activity may have been a consequence of the increased GSH levels. Although extrinsic apoptosis was activated in THP-1 cells, C_LE_ (0.2–0.4 mg/ml) decreased caspase-3/7 activity, indicating that apoptosis was not fully executed (24 h). Interestingly, C_LE_ increased THP-1 caspase (−8, −9, −3/7) activities at 0.8 mg/ml (24 h), suggesting an increased initiation and execution of THP-1 apoptosis.

At 72 h, caspase activities were decreased in both cell lines, suggesting a decreased activation of apoptosis. In PBMC’s and THP-1 cells, the increase in GSH levels and the decrease in caspase (−9, −3/7) activities by C_LE_ (0.05–0.8 mg/ml, 72 h) may have decreased apoptotic cell death. However, PBMC and THP-1 cell viability was deceased at 0.4–0.8 mg/ml C_LE_ and 0.8 mg/ml C_LE_ respectively, suggesting an alternative form of cell death occurred.

Increased caspase-3 and proteasome activity, as well as E3 ubiquitin-conjugating enzyme expression are associated with increased proteolysis [56]. Thus the ability of C_LE_ to down regulate caspase activities in PBMC’s and THP-1 cells may decrease proteolysis and the progression of cancer cachexia.

The cachectic syndrome is characterized by a negative energy balance due to reduced food intake and abnormal metabolism [57]. The inability to ingest/ use nutrients [5] and the negative energy balance present in cachectic patients leads to catalysis of muscle and fat stores for energy production [58]. In PBMC’s, C_LE_ decreased ATP levels, a possible consequence of the decreased cell viability. Cancer cells require high levels of ATP for cellular proliferation [59]. In THP-1 cells, C_LE_ decreased ATP levels which may decrease THP-1 cell proliferation. However in cachexia, a decrease in ATP levels may contribute to tissue wasting.

The potent feeding stimulant neuropeptide Y (NPY) promotes food and energy intake [60]. Increased cytokine (IL-1, IL-6, TNF-α) levels may inhibit NPY signalling leading to decreased food intake and increased energy expenditure [60]. Leptin functions as a suppresser of food intake and stimulator of energy consumption [6]. Pro-inflammatory cytokines may inhibit feeding by mimicking the hypothalamic negative-feedback signalling effect of leptin [61]. Thus, the ability of C_LE_ to decrease pro-inflammatory cytokine levels may increase food intake, decrease energy expenditure and possibly combat the negative energy balance associated with cancer cachexia.

## Conclusion

Our results show that C_LE_ increased oxidant scavenging activity and GSH levels, modulated pro-inflammatory cytokine levels and regulated apoptosis and caspase activity in normal PBMC’s and THP-1 cells. C_LE_ may thus be effective in cancer cachexia.

## Abbreviations

ANOVA: One way analysis of variance
ATP: Adenosine triphosphate
BHT: Butylated hydroxytoluene
*C. asiatica*: *Centella asiatica*
C_LE_: *C: asiatica* ethanolic leaf extract
DMSO: Dimethyl sulphoxide
DNA: Deoxyribonucleic acid
DPPH: 2, 2-diphenyl-1 picrylhydrazyl
ELISA: Enzyme-linked immunosorbant assay
FA’s: Fatty acids
FCS: Foetal calf serum
GSH: Gluthatione
h: Hours
HSL: Hormone sensitive lipase
IL: Interleukin
LPL: Lipoprotein lipase
LPS: Lipopolysaccharide
Min: Minute
NF-κB: Nuclear factor kappa B
NO: Nitric oxide
NPY: neuropeptide Y
PARP: Poly (ADP-ribose) polymerase
PBMC’s: Peripheral blood mononuclear cells
PBS: Phosphate buffered saline
PSF: Pen/Strep Amphotericin B
RLU: Relative light units
ROS: Reactive oxygen species
RT: Room temperature
SA: South Africa
TAG: Triacylglycerol
THP-1: A leukaemic cell line
TNF-α: Tumour necrosis factor-α
WST-1: 4-[3-(4-iodophenyl)-2-(4-nitrophenyl)-2H-5-tetrazolio]-1,3-benzene disulfonate
